# Biliary balloon dilator impaction in a non-dilated bile duct with anatomical variations: a case report

**DOI:** 10.1186/s12876-022-02196-y

**Published:** 2022-03-18

**Authors:** Takeshi Okamoto, Kazuki Yamamoto, Katsuyuki Fukuda

**Affiliations:** 1 Department of Hepato-Biliary-Pancreatic Medicine, Cancer Institute Hospital of Japanese Foundation for Cancer Research, 3-8-31, Ariake, Koto-ku, Tokyo, 135-8550 Japan; 2 Department of Gastroenterology, St. Luke’s International Hospital, 9-1 Akashicho, Chuo-ku, Tokyo, 104-8560 Japan

**Keywords:** Gallstones, Bile ducts, Choledocholithiasis, Cholangiopancreatography, endoscopic retrograde, Case reports

## Abstract

**Background:**

While techniques for extracting large stones from dilated bile ducts are increasing, options for small stones impacted in non-dilated bile ducts are limited.

**Case presentation:**

We report the case of an impacted biliary balloon dilator in a choledocholithiasis patient with a non-dilated bile duct and multiple anatomical variations, including low insertion of the cystic duct. After unsuccessful attempts with a stone extraction basket and balloon, a biliary balloon dilator with a sharp catheter tip was advanced into the bile duct. The balloon could not be removed from the bile duct even when deflated. The duodenoscope fell back into the stomach, causing the shaft of the dilator to break near the ampulla. We then removed the broken tip with a snare, which caused the balloon sheath to separate from the shaft and remain in the bile duct. Finally, we removed the sheath with rat-tooth forceps, leading to successful extraction of the stone-and-balloon complex.

**Conclusions:**

The exceedingly rare possibility of balloon impaction should be kept in mind when using biliary balloon dilators in non-dilated bile ducts.

**Supplementary Information:**

The online version contains supplementary material available at 10.1186/s12876-022-02196-y.

## Background

The use of stone extraction baskets and balloons in endoscopic retrograde cholangiopancreatography (ERCP) has gained widespread acceptance as safe and effective methods for the removal of common bile duct stones. However, over 100 cases of basket impaction have been reported, with an estimated frequency as low as 0.6% in recent reports [1]. Balloon impaction is an extremely unlikely event, as a deflated balloon can usually bypass stones on its way out of the bile duct. Reported non-surgical strategies for impacted basket retrieval, including extracorporeal shockwave and laser lithotripsy, balloon dilation, and use of a second basket, forceps, snare, or rescue mechanical lithotripter, are difficult to apply to impacted balloons in slim bile ducts. Herein, we report a case of an impacted biliary balloon dilator due to stone-and-balloon complex formation in a non-dilated bile duct with anatomical variations.

## Case presentation

A 45-year-old obese Japanese woman presented with abdominal pain of 3 days’ duration. The pain started 3 h after eating sausages and eggs. Her medical history was only notable for uterine fibroids. She was taking no medications, supplements, or herbal remedies and no recent history of sick contacts or overseas travel. She was an occasionally drinker and had quit smoking over 20 years ago. She denied food or drug allergies.

Upon presentation, the patient was in moderate distress. Her vital signs included body temperature of 37.1 degrees, blood pressure of 112/74 mmHg, heart rate of 92 beats per minute, and respiratory rate of 18 times per minute. Rebound tenderness in the right upper quadrant and Murphy’s sign were noted on physical examination. Laboratory results were significant for a white blood cell count of 19,500/mm^3^ (reference range: 2900–7800/mm^3^) and C-reactive protein of 28.9 mg/dL (reference range: < 0.30 mg/dL). Bilirubin and hepatobiliary enzymes were within their normal ranges. Computed tomography led to the diagnosis of acute cholecystitis.

Antibiotics were started and percutaneous transhepatic gallbladder drainage (PTGBD) was performed the next day, with some symptomatic relief. However, the patient complained of severe abdominal pain 2 days after admission. Her body temperature increased to 38.8 degrees Celsius. Repeated laboratory testing revealed total bilirubin of 5.3 mg/dL (reference range: 0.2–1.2 mg/dL), aspartate aminotransferase of 159 U/L (reference range: 9–32 U/L), alanine aminotransferase of 205 U/L (reference range: 3–38 U/L), and alkaline phosphatase of 1118 U/L (reference range: 103–289 U/L).

**Fig. 1:**
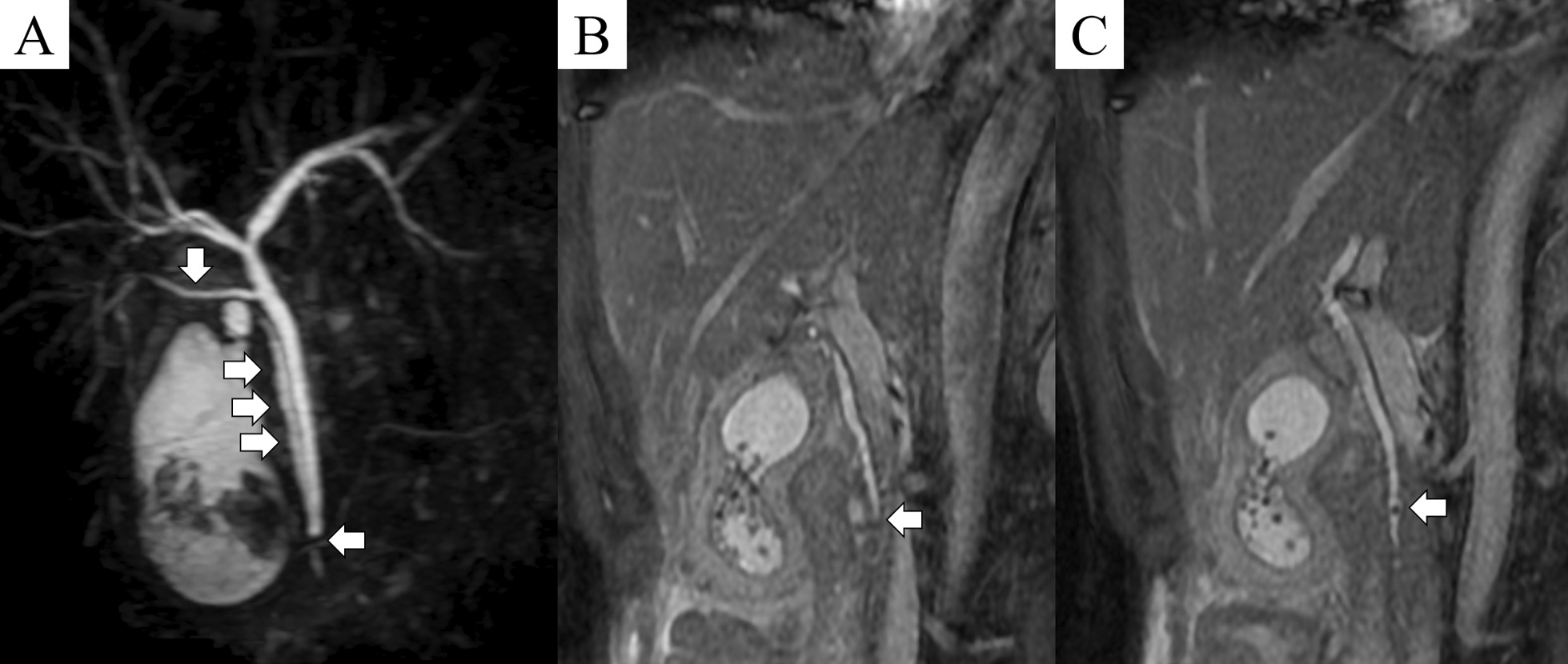
Magnetic resonance cholangiopancreatography. **A** Magnetic resonance cholangiopancreatography revealed 3 anatomical variants of the biliary tree: low insertion of the cystic duct (arrow pointing left), long parallel cystic duct (arrows pointing right), and the right posterior sectoral bile duct flowing directly into the common bile duct (arrow pointing down). **B** No clear stones were found in the cystic duct. The arrow points to the insertion of the cystic duct. **C** A 3 mm stone (arrow) was observed in the distal bile duct (4 mm diameter), immediately below the insertion of the cystic duct

Several anatomical variations were noted on magnetic resonance cholangiopancreatography (MRCP). The cystic duct joined the distal third of bile duct, suggesting low insertion of the cystic duct (LICD). A long cystic duct ran parallel to the bile duct. Furthermore, the right posterior sectoral bile duct flowed directly into the common bile duct (Fig. 1A). A 3 mm stone was impacted in the lower common bile duct (4 mm diameter) immediately below the insertion of the cystic duct (Fig. 1B, C). The patient was diagnosed with acute cholangitis due to an impacted common bile duct stone. The gastroenterology department was consulted.

**Fig. 2:**
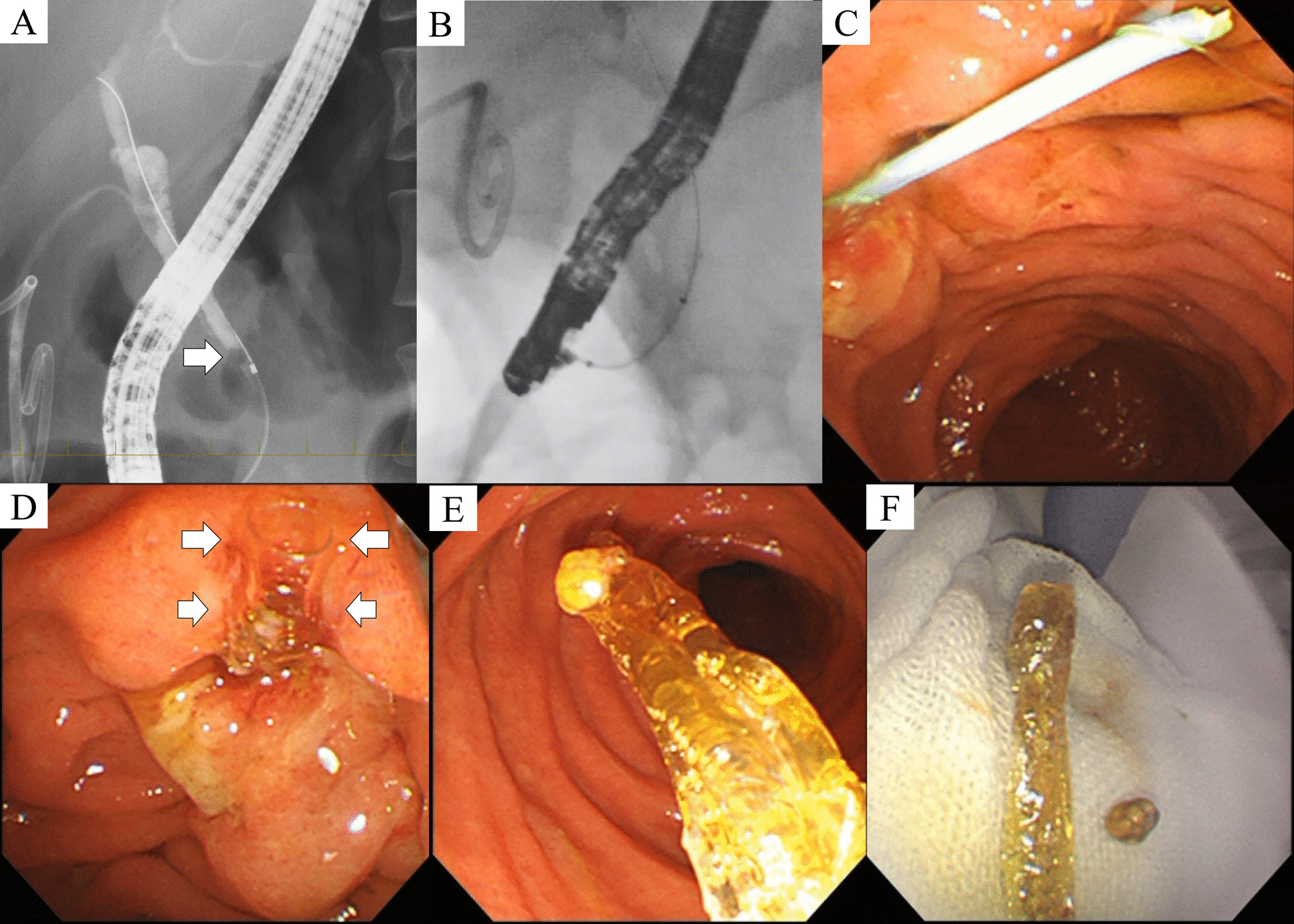
Endoscopic retrograde cholangiopancreatography. **A** Fluoroscopy revealed a small stone (arrow) in a non-dilated bile duct with low insertion of the cystic duct. **B** A biliary balloon dilator became impacted in the bile duct. **C** The shaft of the balloon broke when the scope fell back into the stomach. **D** The shaft slipped out of the transparent balloon sheath (arrows) when pulled with a snare. **E**, **F** The stone-and-balloon complex was successfully removed with rat-tooth forceps

Emergency ERCP was performed using the JF-260V duodenoscope (Olympus Corporation, Tokyo, Japan) (Additional file 1). Bile duct cannulation was achieved with some difficulty, due to LICD and the parallel cystic duct. Fluoroscopy revealed a 5 mm stone in a non-dilated bile duct with LICD. Contrast was also injected from the PTGBD tube, confirming that the cystic duct ran parallel to the bile duct and overlapped with it on fluoroscopy, even when the C-arm was rotated (Fig. 2A).

After endoscopic sphincterotomy, an eight-wire stone extraction basket (MB-35-2X4-8; Cook Medical Inc., Bloomington, Indiana, USA) was selected for stone extraction. After several unsuccessful attempts, we switched to a stone extraction balloon (Multi-3V Plus; Olympus), but the catheter could not be advanced beyond the impacted stone. We therefore used a biliary balloon dilator with a sharp catheter tip (Eliminator PET Biliary Balloon Dilator (8 mm × 3 cm); CONMED Corporation, Utica, New York, USA). As we feared that dilating the balloon in the narrow lower bile duct (where the stone is) could lead to bile duct perforation, we decided to inflate the balloon above the stone, to use it as a stone extraction balloon. The balloon dilator was successfully advanced into the proximal bile duct. When the balloon was inflated above the stone and pulled, the impacted stone did not budge from its initial position. The balloon was then deflated, but still could not be withdrawn from the bile duct (Fig. 2B).

The duodenoscope fell back into the stomach while trying to retract the balloon dilator, causing the shaft of the dilator to break near the ampulla (Fig. 2C). We pulled the broken tip with a snare, which caused the balloon sheath to separate from the shaft and remain in the bile duct (Fig. 2D). We finally retrieved the stone-and-balloon complex by grasping the sheath with rat-tooth forceps (Fig. 2E, F). A 6-French, 100 mm double-pigtail plastic stent (Hanaco Medical Co., Ltd., Saitama, Japan) was inserted at the end of the procedure.

**Fig. 3:**
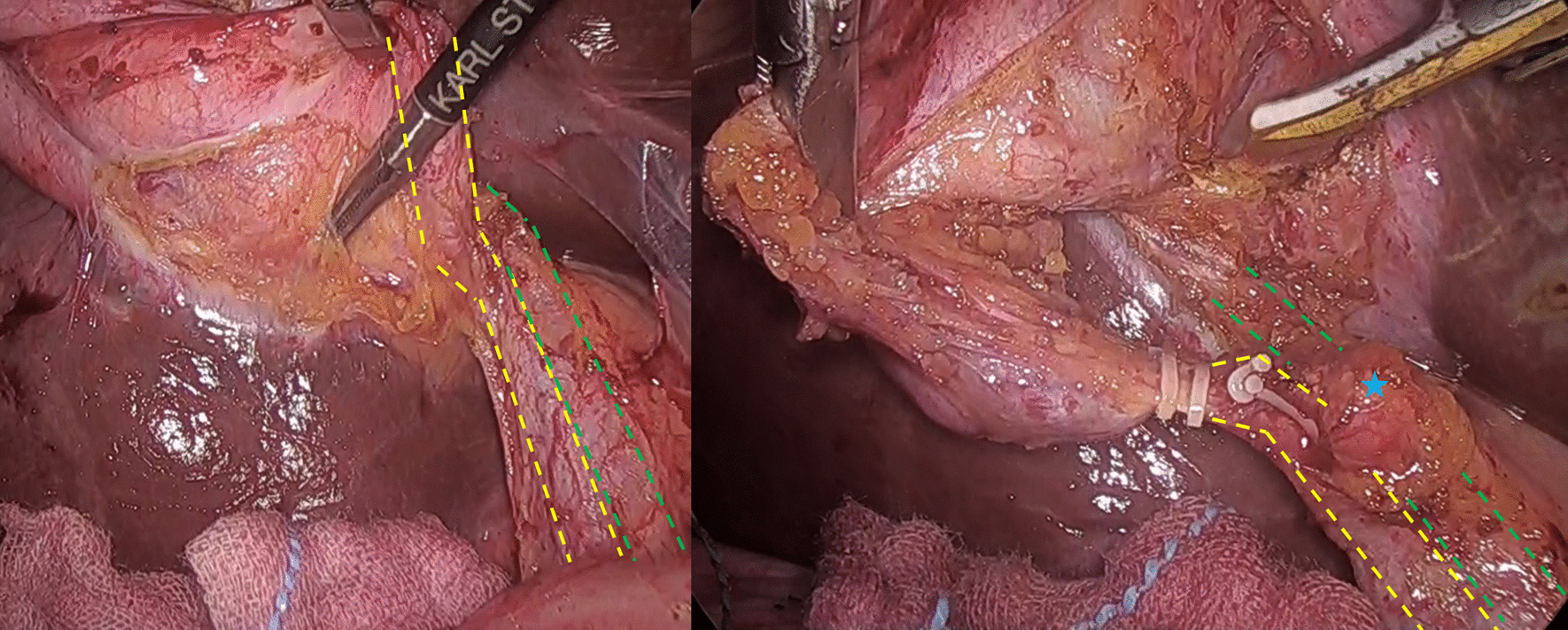
Laparoscopic cholecystectomy. A long cystic duct (dotted yellow lines) running parallel to the common bile duct (dotted green lines) starting from the level of lymph node No. 12c (blue star) was observed under thin connective tissue. Low insertion of the cystic duct could not be confirmed during surgery

No signs of perforation were observed in a follow-up CT taken immediately after the procedure. Analysis of extracted stone revealed that it was composed of pure cholesterol. Laboratory markers showed marked improvement the next day and the post-treatment course was uneventful. The patient underwent laparoscopic cholecystectomy 3 months later, with no complications. During surgery, the long cystic duct running parallel to the common bile duct could be confirmed (Fig. 3). MRCP taken after discharge revealed that 4 cm of the cystic duct remained after surgery. No stones were observed in the bile duct or cystic duct.

## Discussion and conclusions

Large stones are generally considered to be difficult bile duct stones [2]. Stones ≤ 11 mm can generally be removed easily by stone extraction baskets and balloon catheters with similar safety and efficacy, in less than 10 min in over 80% of cases [3]. Other reported risk factors for difficult stones include stricture distal to the stone, small common bile duct/stone diameter ratio, stone impaction, and higher bilirubin [2]. While the bile duct diameter in our case falls outside 2 standard deviations of all studies on difficult bile duct stones to date, it fulfills all of the last 3 criteria.

Bile duct obstruction due to choledocholithiasis typically causes the proximal bile ducts to dilate. However, 3.9% of cholecystectomy patients with minimal bile duct diameters of 4 mm or less were reported to have common bile duct stones in an ultrasound study [4]. No studies have evaluated options for impacted stones in very slim bile ducts.

Anatomical variants of the cystic duct may relate to location of insertion (high/low/medial/anterior/posterior), length (short/long), course (parallel to the bile duct), and drainage (into the right hepatic duct/directly into the duodenum/from the right posterior sectoral hepatic duct) [5]. Imaging studies have shown that LICD, which refers to cystic ducts joining the bile duct at the distal third between the hepatic hilum and ampulla of Vater, occurs in 5.6–11% of patients [5, 6]. Long, parallel cystic ducts occur in 7.5–10.6% of cases and may co-exist with LICD [5–7].

Slim bile ducts with anatomical variations leave the endoscopist with limited options when stones cannot be extracted with baskets and balloons. The bile duct in our case was too thin to allow for the insertion of a second device, drainage tube, or stent. The percutaneous transhepatic approach was also infeasible, as the patient’s intrahepatic bile ducts were also extremely slim (the right hepatic duct was less than 3 mm). The PTGBD route also did not assist in proximal drainage due to LICD and intervention through this route would be extremely difficult given the long cystic duct. Extracorporeal shock wave lithotripsy usually aims to break large stones into fragments of less than 5 mm [8]. Laparoscopic choledochotomy is contraindicated in non-dilated bile ducts, leading stones less than 7 mm to be excluded from laparoscopy studies [9]. As open surgery would have been the only viable alternative, we had little choice but to attempt this off-label use of the balloon dilator.

Unlike stone extraction balloons, biliary balloon dilators cannot revert to their original form once dilated. They remain more spread out in a wrinkled state, often making it difficult to remove from the duodenoscope after use. Therefore, impaction of biliary balloon dilators can theoretically occur, while the chances are much lower for stone extraction balloons. It is also possible that there was injury to the balloon itself, causing the outer sheath to become entangled with the stone.

In addition, the package insert for the Eliminator PET balloon also explicitly warns about a possibility of shaft breakage. In our case, this ultimately worked to our benefit in removing the stone-and-balloon complex. Naturally, intentional breakage is ill-advised. A device which combines the sharp catheter tip of the Eliminator PET balloon with the design of stone extraction balloons may be helpful in extracting stones from slim bile ducts.

Impacted bile duct stones in non-dilated ducts should be approached with caution, particularly in the presence of anatomical variants such as LICD. No research to date has elucidated predictors of difficult stones or methods for stone extraction in this population. Due to the rarity of these conditions, multicenter studies are desirable.

## Supplementary Information

**Additional file 1.** Video of the endoscopic retrograde cholangiopancreatography procedure showing balloon dilator impaction, breakage, and successful removal.

## Data Availability

Not applicable.

## Abbreviations

ERCP: Endoscopic retrograde cholangiopancreatography
MRCP: Magnetic resonance cholangiopancreatography
LICD: Low insertion of the cystic duct
PTGBD: Percutaneous transhepatic gallbladder drainage

## References

[1] O'Brien JW, Tyler R, Shaukat S, Harris AM (2017). Laparoscopic common bile duct exploration for retrieval of impacted Dormia basket following endoscopic retrograde cholangiopancreatography with mechanical failure: case report with literature review. Case Rep Surg.

[2] Usküdar O, Parlak E, Dişibeyaz S, Köksal AS, Çiçek B, Kılıç ZMY (2013). Major predictors for difficult common bile duct stone. Turk J Gastroenterol.

[3] Ozawa N, Yasuda I, Doi S, Iwashita T, Shimizu M, Muka T (2017). Prospective randomized study of endoscopic biliary stone extraction using either a basket or a balloon catheter: the BasketBall study. J Gastroenterol.

[4] Hunt DR (1996). Common bile duct stones in non-dilated bile ducts? An ultrasound study. Australas Radiol.

[5] Sarawagi R, Sundar S, Gupta SK, Raghuwanshi S (2016). Anatomical variations of cystic ducts in magnetic resonance cholangiopancreatography and clinical implications. Radiol Res Pract.

[6] Kao JT, Kuo CM, Chiu YC, Changchien CS, Kuo CH (2011). Congenital anomaly of low insertion of cystic duct: endoscopic retrograde cholangiopancreatography findings and clinical significance. J Clin Gastroenterol.

[7] Pavlidis TE, Triantafyllou A, Psarras K, Marakis GN, Sakantamis AK (2008). Long, parallel cystic duct in laparoscopic cholecystectomy for acute cholecystitis: the role of magnetic resonance cholangiopancreatography. JSLS.

[8] Tandan M, Reddy DN (2011). Extracorporeal shock wave lithotripsy for pancreatic and large common bile duct stones. World J Gastroenterol.

[9] Jinfeng Z, Yin Y, Chi Z, Junye G (2016). Laparoscopic management after failed endoscopic stone removal in nondilated common bile duct. Int J Surg.

